# On Finding and Using Identifiable Parameter Combinations in Nonlinear Dynamic Systems Biology Models and COMBOS: A Novel Web Implementation

**DOI:** 10.1371/journal.pone.0110261

**Published:** 2014-10-28

**Authors:** Nicolette Meshkat, Christine Er-zhen Kuo, Joseph DiStefano

**Affiliations:** Biocybernetics Laboratory, Departments of Computer Science and Medicine and Computational and Systems Biology Interdepartmental Program, University of California Los Angeles, Los Angeles, California, United States of America; University of South Australia, Australia

## Abstract

Parameter identifiability problems can plague biomodelers when they reach the quantification stage of development, even for relatively simple models. Structural identifiability (*SI*) is the primary question, usually understood as knowing which of *P* unknown biomodel parameters *p*_1_,…, *p_i_*,…, *p_P_* are-and which are not-quantifiable in principle from particular input-output (I-O) biodata. It is not widely appreciated that the same database also can provide quantitative information about the structurally unidentifiable (not quantifiable) subset, in the form of explicit algebraic relationships among unidentifiable *p_i_*. Importantly, this is a first step toward finding what else is needed to quantify particular unidentifiable parameters of interest from new I–O experiments. We further develop, implement and exemplify novel algorithms that address and solve the *SI* problem for a practical class of ordinary differential equation (ODE) systems biology models, as a user-friendly and universally-accessible web application (app)–COMBOS. Users provide the structural ODE and output measurement models in one of two standard forms to a remote server via their web browser. COMBOS provides a list of uniquely and non-uniquely *SI* model parameters, and–importantly-the *combinations* of parameters not individually *SI*. If non-uniquely *SI*, it also provides the maximum number of different solutions, with important practical implications. The behind-the-scenes symbolic differential algebra algorithms are based on computing Gröbner bases of model attributes established after some algebraic transformations, using the computer-algebra system Maxima. COMBOS was developed for facile instructional and research use as well as modeling. We use it in the classroom to illustrate *SI* analysis; and have simplified complex models of tumor suppressor p53 and hormone regulation, based on explicit computation of parameter combinations. It’s illustrated and validated here for models of moderate complexity, with and without initial conditions. Built-in examples include unidentifiable 2 to 4-compartment and HIV dynamics models.

## Introduction and Motivation

### Nonlinear Dynamic System Models, Identifiability Notions and Motivational Examples

**Structural identifiability (** ***SI*** **)** analysis addresses the question of whether quantification of model parameters *p*_1,_ *p*_2_, … is possible from a given set of ideal, noise-free input-output (I-O) data [1], [2]. In this sense, it establishes the limits of knowledge attainable about the parameters from real I–O data, and is a necessary condition for quantifying them from data. For this reason, it is sensible to consider *SI* properties of models prior to attempting to quantify them. This is particularly important for modeling in systems biology, where models are typically large – with many parameters – and I–O data are few, e.g. protein network models, among others [1].

For *SI* analysis, no real data are needed – only model topology or structure – which includes initial conditions (ICs) and the locations of model inputs and outputs – together representing a model of a system and an experiment. Identifiability notions have relevancy primarily in the context of I–O experiments. I–O structure must be included explicitly in the statement of the model. The model also must account for all *a priori* information related in any way about the parameters. The *constrained structure* **–** Eq. (1) below – or its equivalent, is a convenient representation of the model for examining *SI* properties [1], [3].

In Eq. (1), ***x*** is an *n*-vector of state variables *x_i_* (e.g. biochemical species); ***p*** is a *P*-vector of unknown parameters *p_i_* (e.g. rate constants); ***u*** is an *r*-vector of test-inputs *u_i_* (e.g. exogenous stimuli); ***f*** is an *n*-vector of nonlinear (or linear) functions of the state, input and parameter vectors (system structure – e.g. stoichiometric equations); ***y*** is an *m*-vector of outputs (e.g. measurements); ***g*** is a linear or nonlinear *m*-vector of output functions of state variables and parameters (output structure); and ***h*** is a *v*-vector of independent constraint relationships involving the parameters (e.g. positivity of rate constants). In these terms, the basic nonlinear ODE **system-experiment model** is:
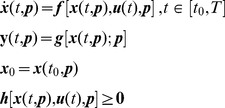
(1)


The four sets of relationships in (1) are the **constrained structure**, the complete model for a system quantification experiment on the interval 0≤ *t* ≤ *T*. Constraints 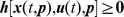 generally affect structural identifiability properties, e.g. ***p***>**0** are common parameter constraints. Identifiability properties are also generally dependent on the form of the input ***u***(*t*), the initial conditions (ICs) 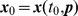, and the period of data observation if the model is nonlinear [2], [3], as in Eq. (1).

In the following developments, we assume the ODE model already has any equality constraints ***h*** = **0** included in the ODEs, e.g. by substitutions. It is convenient to abbreviate the system-experiment model (1) as vector equation: 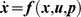, (with equality constraints included), output (vector) ***y*** *=* ***g***(***x, p***) and initial conditions (ICs) 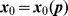. We also assume all parameters ***p***>**0**, the usual way parameters are defined in mechanism-based structural models. Additionally, for the purposes of algorithm developments in this paper, where we apply the differential algebra approach developed in [4]–[6], we assume that ***f*** and ***g*** are rational polynomial functions of their arguments. Notably, rational polynomial function expressions as in mass-action based kinetics – for example – are common in systems biology models.

The following four definitions codify **structural identifiability (** ***SI*** **)** of models and their parameters [7].

1. *The single parameter p_i_* of the constrained structure (1) is structurally **unidentifiable** (abbreviated **unID**) on the interval [*t*_0_, *T*] if there exists an (uncountably) infinite number of solutions for *p_i_* from these relationships. If one or more *p_i_* is (structurally) unID, then the **model** is **unidentifiable**.
2. The *single parameter p_i_* of the constrained structure (1) is **nonuniquely structurally identifiable** **(** ***SI*** **)** on the interval [*t*_0_, *T*] if there exists more than one distinct solution for *p_i_* from these relationships.
3. The *single parameter p_i_* of the constrained structure (1) is **uniquely structurally identifiable** **(** ***SI*** **)** on the interval [*t*_0_, *T*] if there exists a unique solution for *p_i_* from these relationships. If all *p_i_* are uniquely structurally identifiable, the **model** is **uniquely structurally identifiable**.
4. The *single parameter p_i_* of the constrained structure (1) is (structurally) **interval identifiable** on [*t*_0_, *T*] if it is unidentifiable (unID) and a finite interval, dependent on these relationships, exists bounding *p_i_*. If all *p_i_* are structurally interval identifiable, the **model** is (structurally**) interval identifiable**.

#### Globally and Locally Identifiable

**Figure 1:**
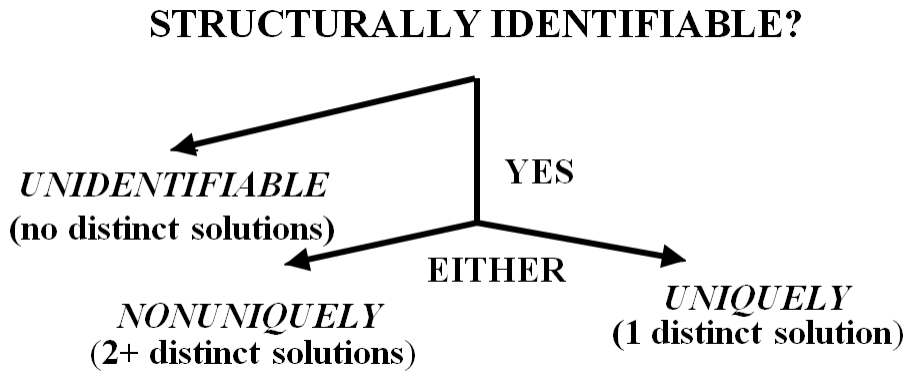
The distinct degrees of structural identifiability *SI* (or unidentifiability) of the parameters and parameter combinations of a dynamic system model.

The adjectives globally and locally are also used in the literature to define some of these notions. **Globally structurally identifiable** **(** ***SI*** **)** simply means uniquely structurally identifiable**. Locally structurally identifiable** or just **identifiable** means structurally identifiable, but not necessarily uniquely. Distinct degrees of identifiability, or lack thereof, are illustrated in ***Fig. 1***.

Also, unidentifiable parameters – although they have an uncountably infinite number of possible solutions – are usually implicitly constrained by the model structure, Relationships (1), which define the interval parameter bounds noted in Definition 4 above. This means they cannot be given any value for, say, model simulation purposes, as explained further in the next paragraph and illustrated in Example 2 below.

#### Identifiable Parameter Combinations and Their Importance

If a model is structurally unID (i.e. if some *p_i_* are not *SI*), there always exist **identifiable combinations** of parameters (abbrev: **combos**), and each such combination can have one or a finite number of solutions. Finding explicit forms of identifiable combinations of parameters in a structurally unID model can be important in model quantification and applications. Combos are *structural invariants* of the model and – for many applications – they can provide a simpler *input-output equivalent* structure, i.e. with the same input-output properties [8], e.g. for simply simulating the model. These structural invariant combos also can provide *interval bounds* for unID parameters, the substance of identifiability definition 4 above [7].

Explicit expressions for *SI* combos in a model can be quite useful, particularly for large nonlinear models that involve tens of state variables and scores or hundreds of parameters – e.g. large protein network models – but also for not-so-large dimensional models. Input-output equivalent models expressed only in terms of *SI* parameters and parameter combos are simpler and therefore easier to implement, quantify and analyze. Explicit parameter combinations also can be used to reveal parameters that might be fixed, or otherwise established independently– an experiment design application – in order to improve identifiability. The algebra, however, can be tedious if accomplished analytically, even for the simplest models. Motivation for automation of this analysis by machine computation is illustrated in the first three examples below – which also illustrate some important results about nonuniqueness of *SI* parameter solutions – further motivating computation of *SI* parameter combinations. Explicit expressions for *SI* combos also can be used to help **reparameterize** a model i.e. fully recast a model in terms of identifiable parameters and parameter combinations, as illustrated in *Example 2* below.

The differential algebra-based algorithms reported by Meshkat and coworkers [4]–[6] are focused here on computing *SI* parameter combinations symbolically, in a new computer program COMBOS, providing *SI* parameter combinations explicitly. COMBOS – described in a subsequent section – is implemented as a user-friendly web application (app), accessible online at: http://biocyb1.cs.ucla.edu/combos.

Three other published computational methods for *SI* analysis of nonlinear ODE models are notable for giving useful information in the unidentifiable case. These include: DAISY, a differential algebra-based method capable of global identifiability solutions [9]. The second method is a generating series approach, called GENSSI – also capable of global identifiability solutions [10]. The third is a non-parametric bootstrap-based algorithm for local identifiability analysis, based on the method of mean optimal transformations [11]. For unidentifiable models, the second and third methods can isolate “groups of functionally related parameters, so that the modeler can determine which parameters must be fixed in order to improve identifiability” [11], but they do not determine algebraic relationships among the parameters. DAISY takes identifiability analysis a step further, and does provide algebraic relationships among the parameters, in a form that can sometimes be manipulated to find *SI* parameter combinations – with varying levels of additional effort – as demonstrated in Examples 1, 3, 5, 6, 8 and 9 below. In contrast, COMBOS completes the task and yields algebraic relationships among *SI* parameters explicitly.

We remark that Examples 1–3 utilize relatively simple linear compartmental ODE models to both illustrate the complexity of the identifiability problems addressed and motivate automated COMBOS solutions for more dynamically complex nonlinear models. These linear examples have analytic solutions and are solvable by classical methods [1], [2], but not without substantial algebraic analysis for achieving solutions – as noted in the examples.

#### Example 1: Unidentifiable 2-Compartment Model (Fig. 2)

This model has been used to represent the dynamics of numerous physiological systems, e.g. distribution and turnover of labeled cholesterol in humans, with impulsive injection and concentration measurements of tracer in compartment 1 [12].
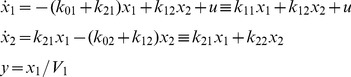
(2)


By definition, *k_ij_* ≥0 for *i* ≠ *j*. We assume *k_ij_* >0 (an additional constraint relation), but there are no known relationships among the *k_ij_*, for *i* ≠ *j*. This model has five unknown nonzero parameters: *k*_01_, *k*_02_, *k*_12_, *k*_21_, and *V*_1_. The right-hand side equalities in the first two equations in (2) are simplified by defining the compartment turnover rates: - *k*_11_ ≡ *k*_01_+ *k*_21_ and - *k*_22_ ≡ *k*_12_+ *k*_02_, which we use in the second part of this example below. The input-output transfer function for this model *H*_11_(*s*) = *Y*(*s*)/*U*(*s*) embodies all information about the parameters that can be obtained from ideal input-output data. *H*_11_(*s*) is evaluated using Laplace transform analysis of the ODEs in (2):
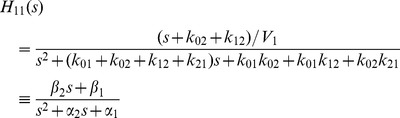
(3)


The *α* and *β* coefficients of the numerator and denominator polynomials 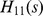 in (3) can always be evaluated uniquely from input/output data (a well-known algebraic property of polynomials) and these are:
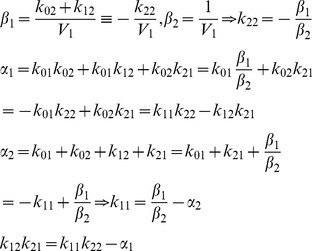
(4)


The *α* and *β* coefficients are thus *SI* parameter combinations (combos). It is also clear from (4) that *V*_1_, 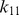, 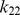 and the product 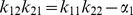
(from 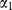 equation in (4)) are another set of four *SI* combinations of this model [1]. In either case, there are five unknown *k_ij_*s in Eqs. (4) that cannot be all found from only these combo equations, and hence the model is structurally unID. Only *V*_1_ can be evaluated uniquely, from Eq. (4).

If it is known that *k*_01_ ≡ 0, a very commonly used *different* 2-compartment kinetic model – e.g. for the disposition of drugs [13] or hormones [14] – we have four unknowns and a *unique* solution is available from Eqs. (4), i.e. the model is uniquely *SI*, because: 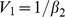, 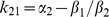, 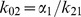 work and 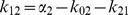. Similarly, if only *k*_02_ ≡ 0, we obtain a (different) model – used even more often to describe drug kinetics, e.g. [15], [16] – and we again have a unique solution for the remaining four parameters [1].

A binary nature for the identifiability (*SI*) question has been illustrated in this example. The model is either uniquely identifiable or it is not identifiable at all, depending on prior knowledge of certain parameters. However, *SI* of all parameters does not generally imply a *unique* solution set, as illustrated in the next example.

#### Example 2: Unidentifiable 2-Compartment Model Reparameterized into Uniquely SI Models

**Figure 2:**
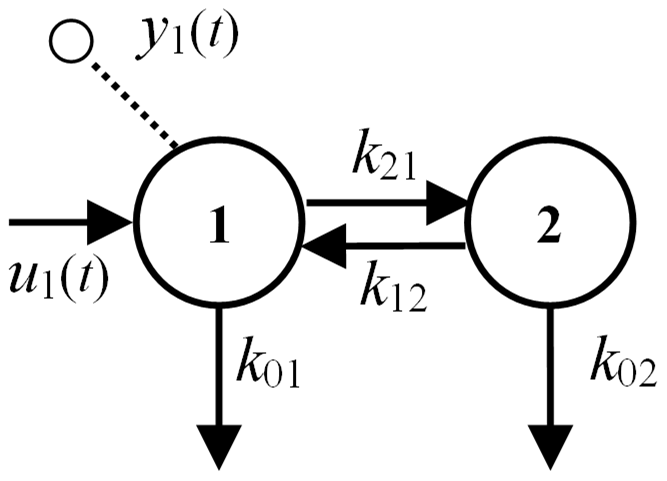
A *SI* 2-compartment model, with both input and output in compartment 1. All *k_ij_* >0 are unidentifiable; but volume *V*_1_ and the three parameter combinations (*combos*) 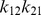, - *k*_11_ ≡ *k*_01_+ *k*_21_ and - *k*_22_ ≡ *k*_12_+ *k*_02_ are uniquely *SI*.

We reparameterize the unidentifiable 2-compartment model in ***Fig. 2*** in two ways here, both in terms of the *SI* parameter combinations. Redefining (*scaling*) the unmeasured state variable *x*_2_ in Eq. (2) as 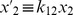 reparameterizes the model with all coefficients as the uniquely *SI parameter combos*: *k*_11_, *k*_22_, *V*_1_ and *k*_12_ *k*_21_.
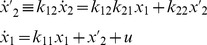
(5)


This model form can be readily simulated. But, it is not a compartmental structure, with balanced exchange fluxes between two compartments and nonzero flux leaks from both.

To preserve the original compartmental structure, we can add and subtract 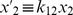 from the 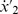 ODE and also add and subtract *k*_12_ *k*_21×1_ from the 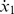 ODE, as in the alternative re-parameterized model:
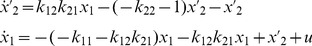
(6)


**Figure 3:**
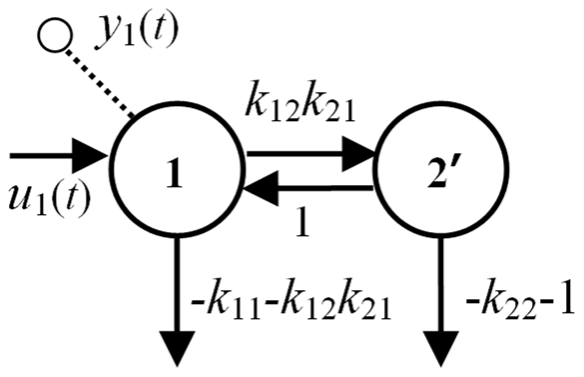
The unidentifiable 2-compartment model of Figure 2 is reparameterized here into a uniquely *SI* equivalent model, with the same structure, and with new parameters evaluated and designated in terms of *SI combos*.

The compartmental model structure in ***Fig. 3*** also has parameters made up only of ID combos. It does, however, have a restricted region of validity, because the leaks in the transformed model must retain their nonnegativity, i.e. 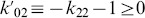 or 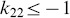 and 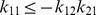, as noted in definition 4 above.

#### Example 3: Some 3-Compartment Models: Combos and Ambiguities

**Figure 4:**
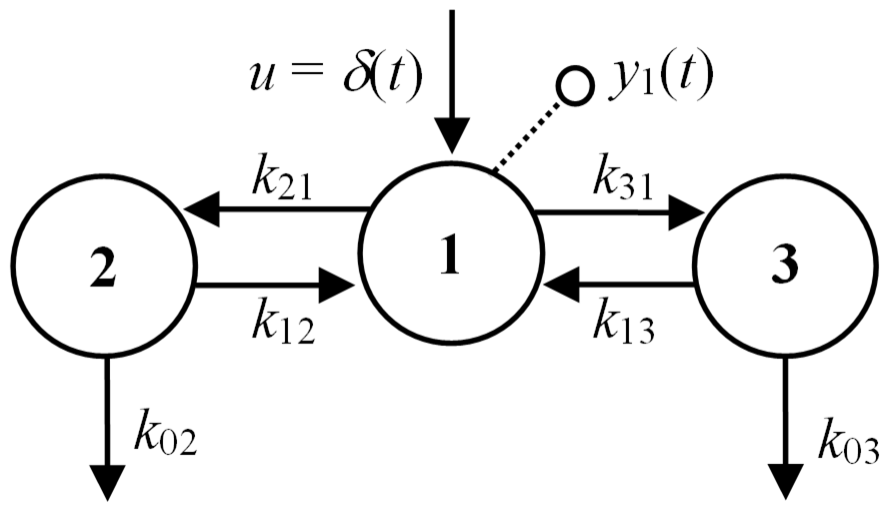
A 3-compartment unidentifiable model with both input and output in compartment 1. None of the *k_ij_* >0 are *SI,* but volume *V*_1_ and the five *combos k*_11_, *k*_22_, *k*_33_, *k*_12_ *k*_21_, *k*_13_ *k*_31_ are uniquely *SI*. If 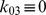, all 5 remaining *k_ij_* become *SI*, but not uniquely – each has *two* feasible solutions.



The model in ***Fig. 4*** is described by:

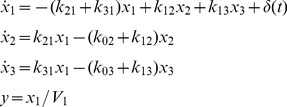
(7)This model has been used, for example, to represent the intra-and extravascular kinetics of thyroid hormone in response to an intravenous tracer dose (impulse *δ*(*t*)) of hormone in the euthyroid human, rat and sheep [17]. It has seven unknown parameters: *k*_02_, *k*_03_, *k*_12_, *k*_13_, *k*_21_, *k*_31_ and *V*_1_, all assumed positive (the constraint relations). The measured output *y*(*t*) is the concentration of substance (tracer hormone in blood plasma) in compartment 1.

The transfer function 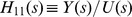 is evaluated from Laplace transforms of Eqs. (7). To simplify the algebra (as in *Example* 1), we define the parameter combinations:
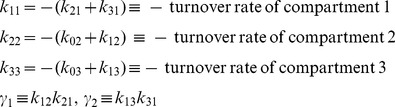
(8)


Then, with much of the detailed algebra suppressed, we arrive at:
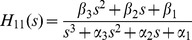
(9)with coefficients evaluated as:



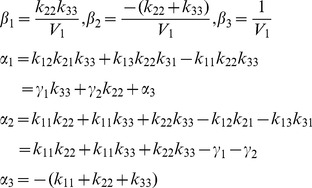
(10)As in *Example* 1, we know all six *α* and *β* coefficients (combo set 1) of the numerator and denominator polynomials of *H*_11_(*s*) can be evaluated uniquely from (ideal) input-output data – Eqs, (10) – over any time interval, i.e. they are uniquely *SI*. The set of combos in Eq. (8), i.e. *k*_11_, *k*_22_, *k*_33_, *k*_12_ *k*_21_, *k*_13_ *k*_31_, plus *V*_1_ in Eq. (10), are also *SI*, but not all uniquely. Either set yields six *k_ij_* equations in the seven unknown parameters. The volume *V*_1_ is uniquely identifiable, in this case directly from Eq. (10), but none of the six *k_ij_* can be determined uniquely from the remaining five equations in Eqs. (8) or (10). We remark that the structural symmetry of the model renders compartments 2 and 3 interchangeable in ***Fig. 4*** ***–*** because input and output are only in central compartment 1 in this example – *the reason* the model cannot be globally identifiable from this limited input-output pair. Indeed, additional algebraic analysis (not shown) indicates that combo *k*_11_ is uniquely identifiable, while combos *k*_22_, *k*_33_, *k*_12_ *k*_21_ and *k*_13_ *k*_31_ are locally ID, each with exactly two solutions – results not easily gleaned by inspection or a less than rigorous analysis.

#### Example 4: Nonuniqueness of SI Parameters and Parameter Combinations

If 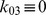 in Eq. (7) we have a different model, one that has been used e.g. to represent bilirubin kinetics [18]. Transfer function analysis yields six independent equations with six unknowns [1]. All parameters of this model *are SI*, but – as in the previous example – not uniquely, despite having 6 equations in 6 unknowns. There are *two* solutions, and both are physiologically feasible if no additional constraints are available *a priori*. The two sets of solutions for all unknown model parameters can be determined from the following algorithm, obtained by successive substitution into Eqs. (7) – (10) with *k*_03_ ≡ 0, i.e.
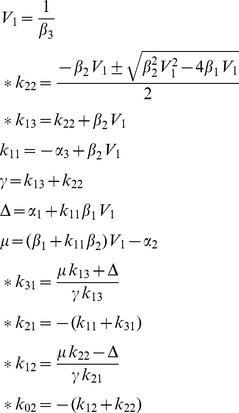
(11)


The equations marked with an asterisk each have two nontrivial combo solutions, because they all involve *k*_22_, which itself has two. Additional information about the model or its parameters is needed to distinguish between these two solutions. Similar results (two solutions) are obtained if only *k*_02_ ≡ 0, yet another variation of this 3-compartment model.

Unidentifiable models in *Examples* 3 and 4 here show how additional parameter information can render a model more identifiable, but not necessarily uniquely. The *Example 3* model has only *SI* combos (and *V*_1_) identifiable, with two distinct solutions for all but one combo, whereas fixing one parameter (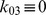) in the same structural model in *Example* 4 renders all remaining individual *k_ij_* parameters *SI*, but still with two distinct solutions.

Besides these complexities, lack of structural identifiability also poses a challenge for further quantitative analysis of these models. For example, without individual parameter or combo values, it cannot be simulated. This indeterminacy can be overcome by reparameterizing the model in terms of the identifiable combinations (combos) of parameters and computing solutions using combos in the ODEs – as illustrated in these motivational examples. Reparameterization, however, requires explicit knowledge of the *SI* combinations and – as also illustrated – the algebra for finding them can be quite tedious. Machine computation clearly is needed to facilitate the process.

## Algorithms, COMBOS Software Implementation and New Results

### Computational Algorithms

We refined and extended the differential algebra algorithms developed for finding *SI* parameter combinations in nonlinear and linear ODE models [4]–[6] and imbedded them in a novel open-source web application (app), COMBOS, all as described below and detailed further in the **Methods** section. This app includes a facile user interface to the open-source computer algebra software package *Maxima* (maxima.sourceforge.net/), implementing the computational engine for COMBOS. We remark that COMBOS finds individually *SI* parameters as well as *SI* combinations.

#### Differential Algebra Algorithms & Extensions for COMBOS

Detailed explanation and proofs of the underlying theory are given in [4]–[6]. In brief, the model ODEs are transformed algorithmically into equations with no state variables, only parameters, inputs and outputs and their derivative terms, using a differential elimination method [6]. The resulting equivalent model is called the *input-output map* 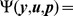 **0**, a subset of a complete *characteristic set*. The mapping from the *P* parameters ***p*** to the coefficients ***c***(***p***) in these input-output equations is tested for uniqueness (distinctness), to determine identifiability, by computing a Gröbner basis for these equations. If this mapping is finite-to-one, the model is identifiable and no additional Gröbner bases are needed and no more are computed in COMBOS – yielding relatively quick solutions. If this coefficient mapping is infinite-to-one, the model is unidentifiable and – in COMBOS – identifiable parameter combinations are computed by finding additional Gröbner bases for the system of equations ***c***(***p***) = ***c***(p*). To speed up computation time, a numerical value is chosen for p*. There are *P*! possible Gröbner bases for the system of equations ***c***(***p***) = ***c***(p*) for a parameter vector ***p*** [4], which can be computationally prohibitive for large *P*. In our earlier work, we generally did not need to find more than *P* different Gröbner bases: trial-and-error randomization of the ordering of the parameter vector ***p*** followed by the computation of Gröbner bases corresponding to *P* shifts of the parameter vector ***p*** generated a sufficient number of *SI* parameter combinations. This worked well enough for small to medium size models, but it does not guarantee that enough combinations would be available for all models. Algorithmic refinements were needed to automate this process in the COMBOS implementation, and this entailed a computability compromise, consistent with algorithmic goals.

The main overall goal is to find *simple* parameter combinations. The simplest identifiable parameter combinations (in terms of polynomial degree and number of terms) among these Gröbner bases is determined by searching them for elements and factors of a certain form, which we call “decoupled” terms [4]. We then add these identifiable parameter combinations to the set of the original coefficients of the input-output equations and determine the simplest algebraically independent set among these. This procedure may not generate the absolute simplest set of identifiable parameter combinations, which would require checking of all *P*! possible Gröbner bases – not practical with current software tools.

Computationally tractable automation of parameter combination selection in COMBOS was implemented by systematic and exhaustive *ordering and reordering* of Gröbner bases, and repeating this process two additonal times – each with a different ordering – to expand the search to a larger subset of the *P*! possible Gröbner bases for the system of equations ***c***(***p***) = ***c***(p*). *3P* was chosen heuristically because it generated a sufficient number of simple identifiable parameter combinations in all example runs of unidentifiable models with as many as 10 parameters.

These algorithmic refinements are implemented in COMBOS as follows. *P shifts* of parameter vector ***p*** are made and Gröbner bases are computed for each of these. The parameter vector is then *reordered* and Gröbner bases corresponding to *P* shifts of this reordered parameter vector are computed. *Shift* means transforming (*p*_1_, *…, p_P_*) to (*p*_2_, …, *p_P_, p*_1_). This gives each parameter a ranking that eliminates it last. *Reordering* means transforming (*p*_1_, …, *p_P_*) to (*p*_1_, *p*_3_, …, *p_P_*_-1_, *p*_2_, *p*_4_, …, *p_P_*) if *P* is even; and (*p*_1_, *p*_3_, …, *p_P_*, *p*_2_, *p*_4_, …, *p_P_*_-1_) is *P* if odd. This gives each parameter a “new neighbor”, so new combinations can possibly be found. This process is repeated one more time, for a total of *3P* rank orderings.

##### Initial Conditions Included

If any initial conditions are known, they are now included algorithmically with the characteristic set and identifiability results are evaluated using the complete characteristic set of equations, as in [19]. Special handling of initial conditions is discussed further below.

### COMBOS: User Interface, Structure & Usage Guidelines

**Figure 5:**
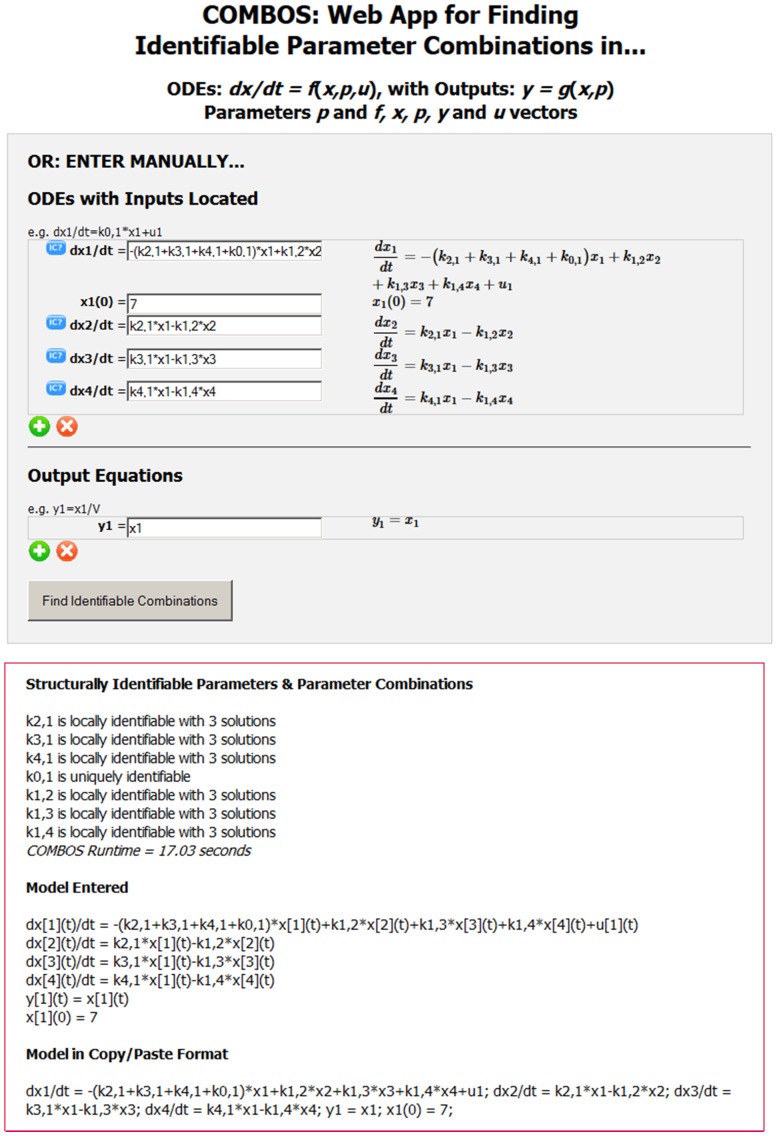
Main features of the COMBOS user interface. (TOP) Header and interactive input of a 4-compartment model example with one output and one IC. Equations entered using programming language are translated on the right into natural math (“pretty”). (BOTTOM) – *SI* analysis results, provided in ∼16 secs. Six of 7 *SI* combos – rendered explicitly – have three feasible solutions each. The **Model in Copy/Paste Format** can be readily used to run variations of this model, by direct editing in the copy/paste input window.

The **Methods** section provides a flowchart of the underlying computational steps involved in COMBOS program interactive use. At the front-end of the user-interface, model equations are entered for *SI* analysis in either of two ways: using a *fill-in form* that prompts for addition of equations, in a standard markup language; or entry into a text area in *copy/paste form*, with equations delimited by semicolons. These modes of model entry circumvent storing user data on the server, thereby minimizing security concerns. Equations are displayed in native math (pretty) form as they are entered, as illustrated in the interface screen shot in ***Fig. 5***, which illustrates computed identifiability analysis results as well as user input. Model equations and initial conditions (ICs) are specified as program input for this 4-compartment model example; and the results show multiple solutions. The standardized syntax is described further below. Note that results also include the model in copy/paste form, which greatly facilitates model reentry, as is, or edited.

#### Syntax & Naming Conventions for Specifying the Model

Equations are entered using ASCIIMathML library notation **(** http://www1.chapman.edu/~jipsen/mathml/asciimath.html), chosen over its MathML and LaTeX counterparts for its simplicity, reduced character count and thus usability. **Table 1** lists the naming guidelines for ODE equations of the form 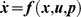, with initial conditions (ICs): ***x***(0, ***p***) and outputs ***y*** *=* ***g***(***x, p***). Users must adhere to this common standardized notation.

**Table 1:** Syntax & Naming Conventions for Specifying the Model.

Time	MUST BE “t”	t
State variables	MUST BE “x” followed by a number	x1, x2 etc
Initial conditions	CAN BE defined for any state variable *x_i_* and MUST BE expressed either numerically or by a function of the parameters *p_j_*. Only functions of parameters in the state equations are allowed.	e.g. 3, 55, x2(0) = 3*p1+ p3
Inputs	MUST BE “u” followed by a number	u1, u2 etc
Outputs	MUST BE “y” followed by a number	y1, y2 etc
Parameters	MUST BE any unreserved letter (NOT x, u, y, t) by itself or followed by any combination of letters, numbers or commas	e.g. p, p4, Vmax, k1,3
Known constants	MUST BE entered as numerical values; otherwise, they are treated as unknown parameters	e.g. 6, 4.32 etc
Operands	MUST BE separated by some combination of operators (+ − */∧) and regular parentheses	e.g. k0,1*x1+ u1

Standardized to facilitate usage and comprehension of results.

Numbering of state variables, inputs and outputs MUST start from 1 and be monotonically increasing. This means the numerical subscript of an input or output does not necessarily correspond to that associated with a state variable (or compartment). For instance, a system with a single output of/from state variable (compartment) 2 is written 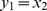; or input *u*_1_ can be in any ODE, not just the first one, etc.

Differential algebra algorithms currently handle only polynomial and rational function terms for *SI* analysis. Terms with trigonometric or transcendental functions, e.g. *sin, cos, ln* – as well as constants (e.g. *pi*, *e*) – are not recognized as such by the parser and would be incorrectly treated as parameters if encountered.

#### Initial Condition Handling in COMBOS

Initial conditions may or may not be known for a given state variable in a model, and knowledge of initial conditions can change the resulting *SI* of the parameters or parameter combinations. The interface provides a convenient way to enter known ICs. Known initial conditions are usually numbers, but – importantly – COMBOS also accommodates functions (combinations) of the parameters, which can be useful in some applications, e.g. when ICs represent nonzero steady state functions of parameters to be estimated from transient response data initiated from steady state (e.g, see [20]). When not known, initial conditions are simply treated as *generic* by default. This means that identifiability properties obtained without initial conditions hold true almost everywhere, i.e. except on a “thin set,” – a set of measure zero [21]. This means there might be *particular* initial conditions that yield different identifiability properties [21]. These are called *inaccessible* states that – under very particular circumstances – lead to unidentifiability of parameters [21]. The COMBOS algorithm presently handles inaccessible initial states for models that have one or more constant ODE solution trajectories, i.e. 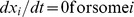, which renders the model *reducible*. These constant solutions are incorporated into the algorithm for finding the input-output (I-O) equations, 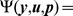 **0,** and change identifiability properties of the model, as illustrated in Example 8. Otherwise the system is assumed accessible from all known initial conditions.

For each state variable, a subfield is available for entering the corresponding initial condition, assumed unknown (generic) and hidden from view at the start, so as not to obscure the view of the overall interface and thus its usability. Visibility of the subfield can be manually toggled so that it is displayed only if there are known initial values to enter. For the copy/paste method, users need only list values for known initial conditions.

For each known initial condition specified, the program checks that none are functions of undefined state variables, inputs, outputs or parameters.

### Application Testing and New Results

#### Comparisons with DAISY

DAISY (Differential Algebra for Idenfiability of SYstems: http://www.dei.unipd.it/~pia) is a freely downloadable computer algebra software package for analyzing parameter *SI* [9]. Users include an input file with the mathematical model in the syntax required by DAISY and run the program using a command line interface. Results provide solutions to the system of parameter-coefficient mapping equations ***c***(***p***) = ***c***(p*) at a *pseudo-random point* p*. In the case of local identifiability, users can then count the number of solutions; and in the case of unidentifiability, they can sometimes algebraically manipulate the solution to get identifiable parameter combinations [4], [5]. In some cases (see *Examples* 5, 6, 8, and 9), this can be readily done by moving all parameters in DAISY solutions to one side of the equations, via addition/subtraction or multiplication/division, but not in general. Even for very simple examples such as the 2- and 3-compartment models of *Examples* 1 and 3 above, this trick of moving all the parameters to one side is not so easy, as we now show.

For *Example 1*– with ***p*** = (*k*_01,_ *k*_02,_ *k*_12,_ *k*_21,_ *V*_1_) – DAISY code generated the pseudo-random point p* = (8, 7, 13, 12, 3) for which it provides precisely the following solution:
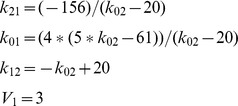



While the *SI* combination *k*_02_+ *k*_12_ is readily obtained from the third equation, by simple rearrangement, the combos *k*_12_ *k*_21_ and *k*_01_+ *k*_21_ are not so easy to unravel. In particular, *k*_01_+ *k*_21_ requires several steps of algebraic manipulation, not so obvious to users unfamiliar with compartmental model equations. The 3-compartment model of *Example 3* requires similar algebraic manipulations to obtain all *SI* parameter combinations. In contrast, COMBOS finds all *SI* combinations explicitly for these examples, in a matter of seconds.

To further evaluate COMBOS functionality and accuracy, we next compared the results of COMBOS *SI* analyses with the example solutions computed using DAISY reported in four papers [9], [19], [21], [22]. Both COMBOS and DAISY provided identical results about *SI* for all of these examples, which included a mix of both linear and nonlinear models up to dimensionality four. We present and discuss only four examples that differed in some respects here, with all models presented in COMBOS math syntax.

#### Example 5

The following 3-compartmental model example [19] has one input and one output, tested with and without initial conditions:
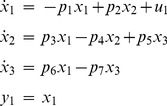
(12)


With *no* initial conditions given, both DAISY and COMBOS indicate the model is unidentifiable. COMBOS, however, additionally indicates explicitly that 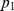 and combination 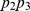 are uniquely identifiable; and 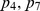 and 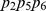 are locally identifiable, with 2 solutions. With additional effort, this also can be derived from the DAISY results, with some additional cross-multiplication computations. As noted in [4], [5], this manipulation does not work for some notable unidentifiable models.

With *all* initial conditions given, both DAISY and COMBOS indicate the model is locally identifiable. COMBOS specifically provides additional *SI* results: the four locally *SI* parameters 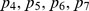 have two solutions (established in 10 seconds runtime). This result also can be attained from DAISY results by counting solutions in the output.

#### Example 6

The following nonlinear polynomial 4-compartmental HIV/AIDS model describes the dynamics of uninfected, latently infected and actively infected cells, along with the free virus particles [22]:
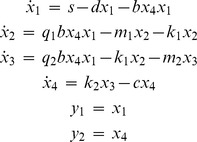
(13)


With no initial conditions given, both DAISY and COMBOS find 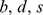 uniquely identifiable and 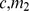 locally identifiable. COMBOS results *additionally* indicate that 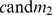 have three local solutions; combination 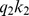 is uniquely identifiable; and 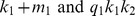 are locally identifiable with*3 solutions* (runtime: 90 seconds). Again, as noted above, this result can be obtained by additional algebraic manipulation of DAISY results.

#### Example 7

This example, from [9], is a 4-compartmental mammillary compartment model with one input and one output, tested with and without initial conditions:
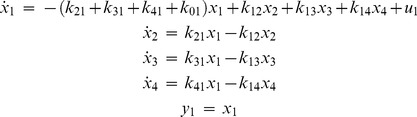
(14)


With no initial conditions given, both DAISY and COMBOS found the model locally identifiable. COMBOS results explicitly indicated that parameter 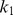 is uniquely identifiable, with the others all locally identifiable – with 3 solutions each. DAISY produces all local solutions, but you have to locate and count them in the output. When at least two initial conditions are known among 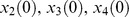, both DAISY and COMBOS results indicate that the model becomes globally identifiable. All COMBOS runs completed in less than 1 minute.

#### Example 8

This simple nonlinear model [21] has one input and two outputs:
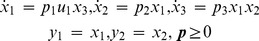
(15)


The authors of [21] use this example to illustrate how DAISY algorithms handle special initial conditions (ICs), i.e. ICs that yield *SI* parameter solutions valid *almost everywhere*. They note that, for partially null (particular) initial conditions 
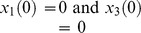
, i.e. {0, *x*_2_, 0}, this model is reducible to 
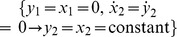
. This means that for this very particular initial state, the model is *inaccessible* from the outputs, for any 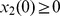, meaning the set of states reachable from these initial conditions is empty. It is noteworthy that this particular IC is an *unstable equilibrium point* of the system, i.e. 
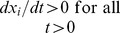
, making it an unlikely IC in practice. In any case, all parameters are clearly unidentifiable – without machine computation. The authors of [21] correctly analyze this special case using DAISY; and also show that for all other initial conditions DAISY finds 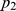 uniquely identifiable.

The COMBOS algorithm also finds all parameters unidentifiable for these particular initial conditions, using a slightly different approach. As noted earlier, COMBOS specifically checks for constant solution trajectories for the initial value problem and incorporates them into the algorithm for finding the input-output equations. In this example, the constant solutions effectively reduce the dimensionality of the input-output relations, thus leading to unidentifiability of the system.

With no initial conditions provided, COMBOS explicitly finds *p*_2_ and combination *p*_1_ *p*_3_ uniquely *SI*. For this simple model, a little extra effort yields the same result from DAISY output, manually extracting *p*_1_ *p*_3_ from the parameter solution for *p*_1_, by cross-multiplication.

All COMBOS runs were completed in about 5 seconds. We remark that, for unidentifiable models in general, DAISY runtimes can be substantially shorter than COMBOS runtimes, because multiple Gröbner Bases are computed in COMBOS in search of identifiable combinations in simplest and explicit form.

#### Benchmarking COMBOS and New Identifiability Results

The dimensionality of models COMBOS can handle is limited by the computational capacity of the underlying symbolic algebraic program, currently Maxima. The first test-group below includes five generic linear models of increasing order *n*, analyzed for *SI* under generic initial conditions. Most of the *SI* parameter combinations results for these models have not previously been published. These examples are then followed by *SI* analysis of several nonlinear systems biology models. The intention is not to give a hard upper bound on the dimensionality that COMBOS can manage, but to show – using systematically more complex examples – that the success of COMBOS is highly dependent on the complexity as well as the size of the model.

**Test-Model 1** is a linear ODE system with a full matrix of 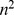 parameters, 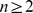, all states measured, one input – in the 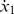 equation only – and generic ICs:
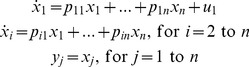
(16)


Results were generated up to dimension *n* = 7, with all parameters found globally identifiable in 3 to 20 secs. COMBOS currently has a default limit of 60 parameters. For *n* = 8, Eqs. (16) have 64 parameters, so computations did not extend beyond *n* = 7. It can be shown by induction that all parameters are uniquely *SI* for any *n*.

**Test-Model 2** is the same linear system, with the same input, but one less output, i.e. 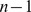 states measured:
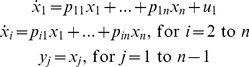
(17)


For this model, it is well-known that *p*_11_ is uniquely *SI* for *n* = 1. For *n* >1, all models are unidentifiable. For *n* = 2, *p*_11_, *p*_22_ and *combo p*_12_ *p*_21_ are uniquely *SI.* For *n* >2, results are more complicated. For example, for *n* = 3, *p*_11_, *p*_21_ and *combos p*_22_+ *p*_33_, *p*_23_/*p*_13_, *p*_22_ – (*p*_12_ *p*_23_)/*p*_13_,

− (*p*_12_ *p*_23_ *p*_33_)/*p*_13_+ *p*_23_ *p*_32_ – (*p*_12_ *p*_22_ *p*_23_)/*p*_13_+ 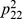 and.

− (*p*_11_ *p*_23_ *p*_33_)/*p*_13_+ *p*_23_ *p*_31_ – (*p*_11_ *p*_22_ *p*_23_)/*p*_13_+ *p*_21_ *p*_22_ are uniquely *SI*, a new result.

**Test-Models 3, 4 and 5** are variations of single-input Test-Model 2– each with different outputs defined. Test-Model 3 has a single output sampled in the same compartment as the input: 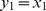. Test-Model 4 has a single output sampled in a different compartment from the input: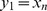. Test-Model 5 has two outputs sampled in separate compartments, with one sample in the same compartment as the input: 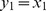 and the second in the *n* ^th^ compartment 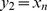.

COMBOS results were generated up to dimension 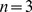 for Test-Models 2 thru 5. Computations did not complete for *n*>3 because the models were too complicated for the Gröbner basis solvers in Maxima. All were found unidentifiable overall, in 3 to 90 seconds runtime. As already noted, some *SI* analysis results for the simplest of these models are consistent with known or published identifiability properties, but most parameter combination results are new and not so simple – as given below.

**For Test-Model** **3**, *n* = 1: *p*_11_ is uniquely *SI*. For *n* = 2: *p*_11_, *p*_22_ and combo product *p*_12_ *p*_21_ are uniquely *SI.* For *n* = 3: *p*_11_ and *combos p*_33_+ *p*_22_, *p*_13_ *p*_31_+ *p*_12_ *p*_21_, *p*_22_ *p*_33_− *p*_23_ *p*_32_ and.

*p*_11_ *p*_22_ *p*_33_ – *p*_12_ *p*_21_ *p*_33_− *p*_11_ *p*_23_ *p*_32_+ *p*_13_ *p*_21_ *p*_32_+ *p*_12_ *p*_23_ *p*_31_− *p*_13_ *p*_22_ *p*_31_ are uniquely *SI.*

**For Test-Model 4**, *n* = 1: *p*_11_ is uniquely *SI.* For *n* = 2: *p*_21_ and *combos p*_22_+ *p*_11_, *p*_11_ *p*_22_− *p*_12_ *p*_21_ are uniquely *SI.* For *n* = 3, *p*_31_ and *combos p*_33_+ *p*_22_+ *p*_11_,

*p*_21_ *p*_32_− *p*_22_ *p*_31_, – *p*_22_ *p*_33_− *p*_11_ *p*_33_+ *p*_23_ *p*_32_+ *p*_13_ *p*_31_ – *p*_11_ *p*_22_+ *p*_12_ *p*_21_ and.

*p*_11_ *p*_22_ *p*_33_− *p*_12_ *p*_21_ *p*_33_− *p*_11_ *p*_23_ *p*_32_+ *p*_13_ *p*_21_ *p*_32_+ *p*_12_ *p*_23_ *p*_31−_ *p*_13_ *p*_22_ *p*_31_ are uniquely *SI.*

**For Test-Model 5**, *n* = 1, *p*_11_ is uniquely *SI.* For *n* = 2, *p*_11,_ *p*_12_, *p*_21_ and *p*_22_ are uniquely *SI.* For *n* = 3, *p*_11,_ *p*_31_ and *combos*: *p*_33_+ *p*_22_, *p*_32/_ *p*_12_, *p*_33_− (*p*_13_ *p*_32_)/*p*_12_, − *p*_13_ *p*_33_+ (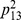 *p*_32_)/*p*_12_− *p*_12_ *p*_23_+ *p*_13_ *p*_22_ and (*p*_11_ *p*_13_ *p*_32_)/*p*_12_− *p*_13_ *p*_31_+ *p*_11_ *p*_22_− *p*_12_ *p*_21_ are uniquely *SI*.

### Moderate Dimension Examples: Reparameterization & Model Reduction

#### Example 9: SI Analysis and Reparameterization of a Minimal Viral Disease Dynamics Model

Equations for a classic viral disease model [23] are rewritten in original form on the left (Target cells *T*, Infected cells *I*, Virions *V*) and in COMBOS form for analysis on the right below. This model has eight unknown parameters: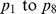. The (constant) input *s* ≡ *p*_1_ *u*(*t*) ≡ *p*_1_ ***1***(*t*) is a step function of unknown magnitude *p*_1_.
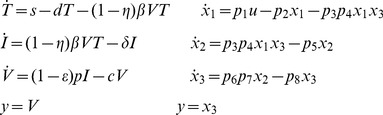
(18)


A slightly simpler version of model (with *s*, *ε* and 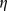 equal zero) was analyzed in [24] for identifiability of parameters *p*_2_, *p*_5_, *p*_7_ and *p*_8_, also using a differential algebra approach. Parameters *p*_2_, *p*_5_ and *p*_8_ were found to be the only parameters identifiable. Analysis using COMBOS online was in agreement, and also provided potentially useful supplemental *SI* information: *p*_2_ and the combos *p*_3_ *p*_4_ and *p*_1_ *p*_6_ *p*_7_ are uniquely identifiable, and *p*_5_ and *p*_8_ are both locally identifiable, each with two solutions. Computation time was ∼10 seconds. Analysis using DAISY provided the same results after algebraically manipulating the parameter solutions.

Using the COMBOS results, the input-output equivalent reparameterized *SI* model (with parameters ***p***) is readily written by scaling *x*_1_ by *p*_6_ *p*_7_ and *x*_2_ by 1/*p*_1_. i.e. 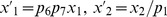. The resulting ODE model is:
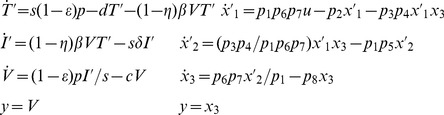
(19)


The structure is identical to the original, but with a new and equivalent set of (structural invariant) parameters: *p*_2_, *p*_5_, *p*_8_ and combos *p*_3_ *p*_4_ and *p*_1_ *p*_6_ *p*_7_.

#### Example 10: Tumor-Suppressor p53 Dynamics Model – Full Nonlinear (NL) Model

This NL model has 4 ODEs, 4 outputs and 23 unknown parameters [1]. The equations in COMBOS ASCIIMathML format are:
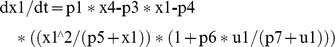





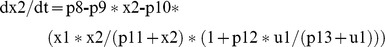





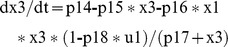





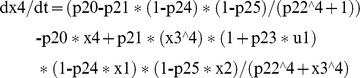


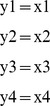



COMBOS, reports all parameters except p22 uniquely *SI*, with p22 locally *SI* with four solutions. Since this model is at least locally *SI*, only one Gröbner basis computation is required. Identifiability results are achieved in about 22 minutes, due to the large number (23) of parameters. However, it is clear that p22, with four possible solutions, has only one nonnegative real – and therefore feasible – solution, because p22 appears only in the single quartic p22∧4 term – in the fourth equation. Therefore *this model is globally identifiable*.

We remark that, whereas the number of nonnegative real solutions is explicitly determined for this particular model, the current implementation of COMBOS does not return a number of nonnegative real solutions in general, because only a single numerical point for p* is tested when solving the equations ***c***(***p***) = ***c***(p*). Testing a different numerical point for p* might lead to a different number of nonnegative real solutions.

#### Example 11: Model Reduction Using COMBOS: Simplifying the p53 Dynamics Model for Facilitating Quantification

The nonlinearities (NL) in this model are of three types: several simple Michaelis-Menten (M-M) functions – of the form: 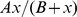, a fourth-order hill function, and products of state variables. For the purpose of model quantification, simplifications of NL terms can be helpful – and were helpful – in quantifying this model from four sets of data corresponding to the output equations above, in [1]. The M–M functions, in particular, are approximately linear (first-order ∼*Ax*) for low substrate values and constant (zero-order combo *A/B*) for large substrate values. Either of these circumstances can in principle occur during transient stimulation of the p53 regulation system by stressor signals and either would therefore be better represented by simpler corresponding M–M terms in the model. In other words, if the substrate signal in an M–M function is very large during a transient, the M–M function is approximately constant. If it is very small, the M–M function is approximately linear. In either case, the number of parameters that need to be estimated is reduced by one.

We systematically screened each of the NL M–M terms in each of the four p53 ODEs, simplifying them one at a time in the above manner, and used COMBOS to delineate the identity of the *SI* parameters and parameter combinations (and their number) resulting from each simplification. For example, the first full ODE:
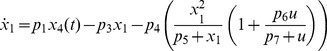
(20)is reduced to the following for (kinase signal intensity) *u* <<*p*_7_, showing the combo *p*_6_/*p*_7_ to be *SI*:



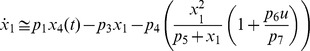



For *u* >>*p*_7_, Eq. (20) is reduced to the following ODE, with 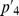 ≡ *p*_4_(1+*p*_6_) *SI*:
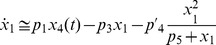



Preliminary quantification of the full (and *SI*) model from experimental data [25] provided initial parameter estimates consistent with inequality *u* >>*p*_7_ and others like it in the four equations. This led to several simplifications, thereby eventually providing more precise parameter estimates for the smaller number of them remaining [1].

## Discussion

Novice modelers may not even recognize that poor identifiability properties are often the problem when model quantification from input-output data fails. Unfortunately, prior structural identifiability analysis of an ODE model can be quite difficult – particularly if it is nonlinear. The good news is that solving identifiability issues has become computationally more practical over the last decade with the advent of various numerical and symbolic computational methods e.g. [4], [9], [11], [26] for addressing various aspects of the nonlinear problem. Symbolic differential algebra approaches have certain advantages, but also significant limitations in practice – due to the nature of symbolic computation. The computational issues remain large – but so does accessibility to this complex methodology. Internet application (app) COMBOS has been developed to overcome the accessibility issues and incrementally advance the computational ones. In addition to the readily accessible, testable and easily modifiable examples built into COMBOS, we have presented 11 additional examples here to further motivate and validate the COMBOS app, and – importantly – to illustrate its utility for modeling and new experiment design – a key subsidiary application of *SI* analysis.

The Saccomani-Bellu-Audoly-D’Angiò group have contributed significantly to both the theory and practice of *SI* methodology and have made available the downloadable DAISY package for *SI* analysis of nonlinear ODE models [9]. A major benefit of the COMBOS and DAISY approaches is that parameter identifiability can be determined *globally* instead of only *locally*, a limitation of other approaches, e.g [11], [27]. Our group has also recently contributed to rendering differential algebra algorithms for *SI* more complete and computationally efficient [4]–[6]. In the current paper, we further update and exemplify these algorithms and present the novel web application COMBOS, which explicitly provides a more complete identifiability analysis than does DAISY [9] and software based on other approaches [11], [26]. DAISY and other packages primarily provide *SI* analysis of individual parameters; COMBOS is designed to do more.

### Features and Limitations of COMBOS

COMBOS is universally accessible, uniquely for this kind of analysis. Nothing needs be downloaded. Users simply access the app via their computer or device browser and run the latest version. All user interaction occurs in her/his local browser. All mathematical computations are done on a remote server maintained by the Computer Science Department at UCLA, with updating of the algorithms and code done by the authors and lab associates of this research team, without the need for local or remote user intervention. In contrast, DAISY, GENSSI and other available software tools require downloading of program files, along with the numerous encumbrances of getting them to function effectively on different local user platforms – typically with dependence on other packages e.g. Matlab for GENSSI, Reduce for DAISY.

The COMBOS interface provides very easy entry of user models, either interactively or in copy/paste form. Natural math language (pretty) feedback of equations entered is provided, for easy checking. Model editing can be done right on the screen.

COMBOS algorithms provide a direct solution approach for finding *SI* parameter combinations – as well as individual *SI* parameters – in otherwise unidentifiable models, bringing the quantification problem closer to resolution. It also provides an upper bound on the number of non-unique solutions for *SI* parameters and parameter combos.

COMBOS computational results are explicit and clear: a list of all parameters *and* parameter combinations that are uniquely identifiable or locally identifiable, along with the maximum number of local solutions. This makes simulating a model easier, from knowledge of *SI* parameter combinations, and provides critical information for subsequent experiment design for quantifying otherwise unidentifiable parameters, by zeroing in on particular ones in combinations that may be *SI* in a different input-output experiment configuration.

For these reasons – in addition to its facile use as a modeling tool – COMBOS is particularly useful for teaching and further research on this subject and we offer it primarily for these purposes.

COMBOS has limitations. For practical computational reasons, COMBOS does not yet distinguish among real and imaginary or positive and negative solutions, so this task remains for the user. But this might not be too difficult, as illustrated for the p53 model example – readily shown to be globally *SI* – with only one feasible (real and positive) solution to a quartic equation involving an otherwise locally *SI* parameter. Future algorithmic enhancements will hopefully resolve this limitation.

The COMBOS interface handles initial conditions facilely, but differently than DAISY algorithmically. When no initial conditions are given, COMBOS and DAISY results are consistent, i.e. they provide identifiability results assuming “generic” initial conditions. When initial conditions are provided, COMBOS and DAISY results are consistent if the system is accessible from those initial conditions. Accessible means that the set of states reachable (at some finite time) from the particular initial condition is not empty [21]. A model might be identifiable without initial conditions known or specified. But fixing *certain* initial conditions, e.g. (0,…,0), could render it unidentifiable. Currently, COMBOS only checks for the case when one or more solution trajectories are constant and the model is reducible, as in *Examples* 1, 3 and 4 of [21]. However, reducible models with nonconstant solution trajectories that remain inaccessible do exist, as in *Example 2* of [21]. This is a weakness of COMBOS; it does not handle all inaccessible initial state situations correctly at this time.

COMBOS performance depends in large part on the computational capabilities of Maxima, the symbolic algebra package in which COMBOS algorithms are implemented. Practically speaking, COMBOS is limited in the dimensionality of problems it can handle, perhaps more limited in this sense than DAISY and other packages. Because COMBOS explicitly provides identifiable parameter combinations, the algorithm is more complex than that in DAISY and others. Only one Gröbner Basis solution is needed in DAISY – for example – to determine individual *SI* parameters. For some cases, by manipulating DAISY output, this is sufficient to also determine identifiable parameter combinations. However, we previously demonstrated, with examples, that this kind of manipulation does not always provide identifiable combinations [4], [5]. In contrast, Gröbner bases of model attributes are computed multiple times to compute identifiable parameter combinations in COMBOS. Specifically, for *P* parameters, *3P* Gröbner Bases are calculated, which can be very computationally time consuming. This algorithmic enhancement, which we found works well in practice, is more efficient than computing and sorting all *P*! possible Gröbner bases. But it still can take a long time to process, depending on model complexity – the cost for this additional information.

These algorithmic enhancements thus limit the size and complexity of models the program can handle. For models too large or complex, COMBOS computations will not finish and the process may run on until it is aborted by the user or the program. A user abort button appears during computations in COMBOS. In some moderate size example runs (e.g. for a 6-dimension thyroid hormone dynamics model [20]), the differential elimination step – to determine the input-output equations – did not complete after many hours of running. For some such models, Maxima either runs out of memory, or the calculation exceeds some internally allowed computation time, and the process aborts. It should be noted, however, that computations for this model did not finish in DAISY either. Thus, the input-output equations in some models – for some still undiscovered reason – may not be amenable to solution by differential algebra methods and both COMBOS and DAISY can fail in this step.

In summary, COMBOS is a readily accessible internet application tool for structural identifiability analysis of linear or nonlinear ODE models with commonly found polynomial or rational function terms, models not “too complex” – of small to medium size. This is a broad class of models in applications, albeit limited in dimensionality for the present.

The subject of identifiability is highly technical and also difficult to comprehend and teach. COMBOS is particularly useful for teaching and further research on this subject and we offer it in large part for these purposes – from any location with an internet connection. The built-in and easily accessible model examples should go far toward teaching upper division and graduate level university students about the basics of structural identifiability concepts and applications, at any location with an Internet connection. And it’s accessible using widely available browsers – on desktops, laptops and even recent smartphones and tablet devices. There is nothing to download and setup by users – a very major enhancement to accessibility of very complex computational solutions to key modeling quantification problems. COMBOS is certainly limited in several respects – but a step forward in this difficult research domain. In the research arena, when COMBOS results explicitly produce complicated parameter combinations, e.g. products of two or three parameters that are identifiable, this is useful “extra” information because it enables users to see which parameters would have to be known *a priori* for the others to be identifiable. This informs new experiment designs, because other I–O pairs are often capable (in principle) of singling out *SI* parameters for estimation.

Well-equipped laboratory toolboxes for solving the largest class of structural identifiability analysis problems should include COMBOS and DAISY, plus other packages – including GENSSI [10], each fulfilling what the other may not for particular problems. The virtually immediate accessibility and facile interface of COMBOS via a user’s web browser renders COMBOS likely to be first on the user test list.

## Methods

### Website

The COMBOS web application implementing the algorithms herein (Version 1.2) is accessible online at: http://biocyb1.cs.ucla.edu/combos. The intention is to keep it updated with new versions as the algorithms are improved.

### Technical Implementation

COMBOS is run on a virtual Apache PHP web server hosted by the UCLA Computer Science Department. It is written in HTML/CSS, PHP and Javascript/JQuery. Equations are dynamically displayed in native math form with MathJax 2.0 and parsed with a PHP script. Maxima symbolic algebra code is run on the parsed input. MathJax 2.0 (http://www.mathjax.org) is an open-source Javascript library that displays ASCIIMathML, MathML and LaTeX markup in native math form on all modern web browsers.

**Figure 6:**
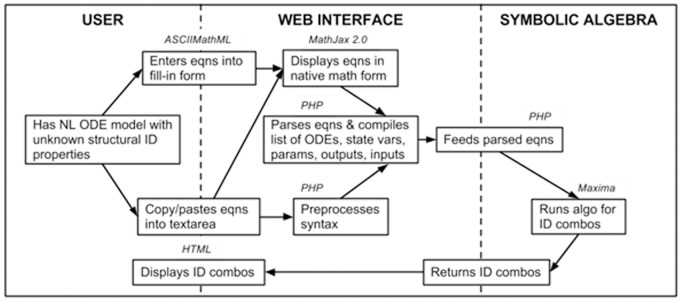
Flow chart of information flow and programming languages used in COMBOS. HTML and ASCIIMathML are utilized in the User section; the Web Interface is written in PHP and MathJax 2.0; and the symbolic algebra is done in Maxima, ported via PHP.

The main parser, written in PHP, is responsible for string parsing, script execution and display of HTML content. ODEs, output equations and initial conditions are stored in separate arrays. The parser loops through the entries in the order listed, simultaneously extracting relevant information and constructing the syntax-appropriate input to Maxima. For each ODE and output equation, regular expressions are used to identify and record the state variables, inputs, outputs and parameters. Parameters are mapped from user-defined to general symbols, to make further data analysis more systematic. After all entries are parsed into a complete Maxima command, the model is run in Maxima and identifiability results are generated. Results displayed include the *SI* parameters and parameter combinations, including the number of nonunique solutions where appropriate. They also include the model with copy/paste equivalent code – useful for modifying the model and rerunning the *SI* analysis; and the parameter mapping table – to help clarify the analysis. ***Fig. 6*** illustrates this information flow in COMBOS.

### Program Documentation and Code

Free source code and license can be obtained by request from the third author (joed@ucla.edu).

### List of Scripts

- **index.php** is the main browser webpage containing the HTML form where users enter equations and where results are displayed.
- **helper2.js** contains Javascript functions that enable: addition and deletion of input fields; fills in corresponding input fields upon clicking example links; toggles the visibility of the initial condition form fields; and calls MathJax.js to display user input in native math (*pretty*) form.
- **upload.php** is the PHP script executed on the browser webpage upon entering a model in copy/paste form. It preprocesses the text, parsing entries separated by semicolons into ODEs with inputs, output equations and initial conditions, and adding the entries to the $_POST array.
- **parser.php** is the PHP script executed on the browser webpage upon form submission. It is either called directly by the fill-in form or indirectly by upload.php. The parser converts all input equations to Maxima markup; maps all user-defined parameters to numbered parameters *p_i_*; creates separate lists for the differential equations, state variables, output variables, input variables, parameters and initial conditions; runs the Maxima code on the lists; and displays Maxima results on the browser webpage.
- **MathJax.js** (v 2.0) is a third-party Javascript package that displays mathematical expressions written in AsciiMathML, LaTeX and MathML markup on all major browsers in native math (*pretty*) form, fixed to make it even “prettier”, because MathJax interprets all multiplication signs (asterisks,*) as dots. To eliminate this extraneous notation, dots are replaced by spaces. This gives the desired aesthetic without modifying the input fed into Maxima.
- **style.css** is the CSS stylesheet used on the main browser webpage.
- In the../files directory: **JSON files** are on the path of several Example links on the main browser webpage of COMBOS. They contain the equations that fill the input fields when an Example link is clicked.
- In the../maxima directory: **FindCombos.maxima** is the Maxima code implementing the symbolic algorithms for finding the identifiable parameter combinations.

### Internal dependencies

./helper2.js.

./files/2CompModelInput.js.

./files/2CompModelIDInput.js.

./files/3CompModelInput.js.

./files/3CompModelICInput.js.

./files/4CompModelInput.js.

./files/4CompModelICInput.js.

./files/HIVModelInput.js.

./parser.php.

./upload.php.

### External dependencies

jQuery (http://jquery.com/).

Mathjax (http://www.mathjax.org/).

Maxima (http://maxima.sourceforge.net/).
